# Prescriptions and Dispensing

**Published:** 1908-08-15

**Authors:** 


### THERAPEUTICS.

### PRESCRIPTIONS AND DISPENSING.

### POWDERS.

POWDERS may be prescribed for use in several ways. The commonest requirement is for internal administration, the mixing of the powder with some suitable vehicle being left to the patient. In other cases one powder is to be taken with each dose of a mixture, the material of the powders being then usually such as reacts with an ingredient of the mixture, causing effervescence for example. Sometimes two powders are prescribed, to be mixed in water by the patient, as in the case of an ordinary Seidlitz powder. Powders may, however, be prescribed for external use also, as dusting powders, etc., or for local application—for example, to the throat by blowing the powder on with bellows, or for snuffing up the nose, and so forth; these latter are usually termed *insufflations* and *snuffs*. When required for swallowing, powders are now often given in the special form of *cachets*.

It will readily be apparent to anyone who has followed what has been said about mixtures and pills that considerably less difficulty may be expected in the dispensing of powders, since most of the trouble encountered in the other cases is due either to physical immiscibility or to chemical incompatibility among some of the ingredients. Powders being necessarily in the same physical state can hardly be immiscible, while chemical action is not very likely to occur between dry substances. Reaction, however, does take place occasionally, and one should always be on the lookout for it.

When considering in general terms the requirements of good dispensing, we gave the second place in order, though not in importance, to the accurate division of the medicine into equal doses; in dispensing such powders as present no special difficulty such accurate division becomes the chief consideration; it includes the mixing of the ingredients into a perfectly homogeneous compound. A few simple rules must be observed to this end. Thus, powdering and mixing simultaneously should never be attempted. If a substance in crystals, granules, or coarse powder is to be mixed with a fine powder, it should itself be ground down to fine powder before mixing.

If a very small quantity of one substance is to be mixed with a much larger quantity of another, the latter should be added in small quantities at a time to the former, each addition being thoroughly mixed in before the next is made. Never try to mix two very unequal quantities of powder by putting the whole of each into one mortar and simply triturating together. For instance, with the following prescription:—

℞ Acidi Arseniosi ... ... ... ... gr. j.
Pulveris Sacchari albi ... ... ... ʒj.
Misce. Divide in pulveres xx.

The wrong way to dispense it is to put the whole of the two ingredients into a mortar and triturate. The arsenious acid must be mixed with two or three grains of the sugar very thoroughly; then three or four grains more sugar added and thoroughly mixed in, and the remainder of the sugar added in a similar way in gradually increasing quantities.

Such a powder as the above raises the question of how the mixing is best done; whether in a mortar with a pestle, or on paper with a spatula. There is a great deal to be said for the latter method, and in some cases it is certainly the better, particularly when friction and consequent heat-production would have a deleterious effect on any of the ingredients. If carefully and thoroughly performed, mixing on paper is probably as good in almost every case as mixing in a mortar when only a few grains are to be dealt with. In the case given above it will be best to mix in the first quantities of sugar both by trituration and on paper, and the final quantities in the mortar only.

With some powders the friction that is sure to arise from the use of pestle and mortar is not merely disadvantageous, but even actually dangerous. Potassium chlorate, for instance, mixed with sugar or other organic and easily oxidisable substance, gives a mixture which the heat generated by friction may cause to explode.

It is often best to divide one ingredient and mix portions of it with others separately, finally mixing the powders so obtained. For instance, in the following prescription:—

℞ Strychninæ ... ... ... ... gr. $\frac{1}{12}$

Elaterini ... ... ... ... ... gr. $\frac{4}{32}$

Bismuthi Subnitratis ... ... ... gr. ij.

Pulveris Cinnamomi compositi ... gr. x.

Fiat pulvis j. Mitte tales lx.

the method to be adopted will be: Weigh out 2 grains of strychnine and powder very finely in a mortar; weigh out 62 grains of compound cinnamon powder, and add it little by little to the strychnine, mixing in the first few grains of it both by the spatula upon paper and by trituration in the mortar, the latter portions by trituration only. When the mixing is complete, weigh off 60 grains of this mixture, setting these aside, and rejecting the remaining 4 grains. Then weigh out $2\frac{1}{2}$ grains of elaterin and 60 grains of compound cinnamon powder, mix these thoroughly together in the same manner, and set aside. Then weigh out 120 grains of bismuth subnitrate and 482 grains of compound cinnamon powder, mixing about a third of the latter with the bismuth salt. Then mix the diluted strychnine thoroughly with the diluted elaterin; add the diluted bismuth, mixing it in thoroughly; and lastly the remainder of the compound cinnamon powder. Each dose of the finally mixed powder will now contain exactly the right proportions of each constituent. With a perfectly homogeneous mixture accurate subdivision is usually a matter of careful weighing merely.

Very small quantities, however, may be divided almost as accurately by trusting to the eye, and the limitations of dispensing weights, and of the accuracy of dispensing scales, sometimes compel one to resort to the eye. For instance, in the above prescription as written, each powder should weigh $12\frac{7}{96}$ grains; as dispensed the total weight is $724\frac{1}{2}$ grains, giving $12\frac{3}{40}$ grains for each powder. The only plan here is to weigh out 12-grain powders and divide the small quantity that remains over as accurately as possible among all the powders.

